# Comparison of Estimated No Surprises Act Qualifying Payment Amounts and Payments to In-Network and Out-of-Network Emergency Medicine Professionals

**DOI:** 10.1001/jamahealthforum.2022.3085

**Published:** 2022-09-16

**Authors:** Erin Lindsey Duffy, Adam Biener, Christopher Garmon, Erin E. Trish

**Affiliations:** 1 University of Southern California Leonard D. Schaeffer Center for Health Policy and Economics, Los Angeles; 2 Economics Department, Lafayette College, Easton, Pennsylvania; 3 Department of Public Affairs, University of Missouri–Kansas City, Kansas City

## Abstract

**Importance:**

The No Surprises Act (NSA), which took effect on January 1, 2022, applies a qualifying payment amount (QPA) as an out-of-network payment reference point. An understanding of how QPA measures compare with the in-network and out-of-network payments physicians received before the NSA implementation may be useful to policy makers and stakeholders.

**Objective:**

To estimate the QPA for geographic and funding markets and compare QPA estimates with in-network and out-of-network payments for 2019 emergency medicine claims.

**Design, Setting, and Participants:**

This cross-sectional study of US commercial insurance claims assessed the Health Care Cost Institute’s 2019 commercial professional emergency medicine claims (*Current Procedural Terminology* [*CPT*] codes 99281-99285 and 99291) and included enrollees in commercial health maintenance organizations, exclusive provider organizations, point of service, and preferred provider organizations self-funded and fully insured through Aetna, Humana, and some Blue Health Intelligence plans. Claims with missing or inconsistent data fields were excluded. Data were analyzed November 1, 2021, to April 7, 2022.

**Main Outcomes and Measures:**

The QPA was calculated as the median allowed amount of all observed claims within strata defined by geographic region, *CPT* code, and funding market. For each stratum, the ratio of mean in-network allowed amounts to QPAs and mean out-of-network allowed amounts to QPAs were calculated. Then the volume-weighted mean of these ratios was computed across *CPT* codes within each geographic and funding market stratum.

**Results:**

The analytic sample included 7 556 541 professional emergency claims with a mean (SD) allowed amount of $313 ($306) and mean (SD) QPA of $252 ($133). Among the 650 geographic and market strata in the sample, the mean in-network allowed amounts were 14% (ratio, 0.96) higher than the estimated QPA. For the subset of strata with a sufficient sample of out-of-network claims (n = 227), the mean out-of-network payments were 112% (ratio, 2.12) higher than the QPA. More generous out-of-network payments were from self-funded plans (120% [ratio, 2.20] higher than the QPA estimate) vs fully insured plans (43% [ratio, 1.43] higher than the QPA estimate). Mean in-network allowed amounts for nonphysician clinicians were 4% (ratio, 1.04) lower than the QPA, whereas mean in-network allowed amounts for physicians were 15% (ratio, 1.15) higher than the QPA estimates. These differences remained after adjusting for geographic region.

**Conclusions and Relevance:**

The findings of this cross-sectional study of US commercial insurance claims suggest that the NSA may have heterogeneous implications for out-of-network payments and negotiating leverage experienced by emergency medicine physicians in different geographic markets, with the potential for greater implications in the self-funded market.

## Key Points

**Question:**

How do qualifying payment amount (QPA) estimates under the No Surprises Act (NSA) compare with in-network and out-of-network payments for emergency medicine services before NSA implementation?

**Findings:**

In this cross-sectional study of 7 556 541 US commercial insurance claims, mean in-network and out-of-network payments were 14% and 112% higher than QPA estimates, respectively. Mean out-of-network payments were higher among self-funded plans than fully insured plans and among physicians vs nonphysicians.

**Meaning:**

The NSA may have heterogeneous implications for out-of-network payments and negotiating leverage for emergency medicine clinicians across geographic markets, plan funding type, and clinician type.

## Teaser

This cross-sectional study compares in-network and out-of-network payments with qualifying payment amount estimates to assess potential implications of the No Surprises Act on private insurance reimbursements for emergency medicine clinicians.

## Introduction

Before implementation of the No Surprises Act (NSA), patients often encountered out-of-network physicians in unexpected situations (eg, an out-of-network emergency or ancillary physician at an in-network hospital), followed by surprise medical bills for the balance of the physician’s charge not covered by insurance. The NSA, which took effect January 1, 2022, banned most surprise medical bills, limiting patient cost sharing to what would be required for an in-network service.^1^ The NSA also established an independent dispute resolution process to determine the insurer’s reimbursement to the out-of-network clinician when the insurer and clinician cannot reach an agreement.

The NSA’s independent dispute resolution process uses final offer arbitration, in which the arbiter must choose either the insurer’s or the clinician’s offer. The federal agencies tasked with implementing the NSA issued an interim final rule in July 2021 that instructed arbiters to consider a qualifying payment amount (QPA), defined as the insurer’s median in-network rate for a service in the NSA-defined region in 2019, adjusted for inflation with the Consumer Price Index for All Urban Consumers.^2,3^ In a second part of the interim final rule issued in October 2021 as well as further guidance issued in April 2022, the agencies clarified that, although arbiters must consider the QPA, they may also consider certain additional information submitted by any party. Allowable information includes the market share of both parties, patient acuity, clinician characteristics, and demonstrations of good faith efforts (or lack thereof) to contract between the parties during the previous 4 years.^3,4^ Arbiters are prohibited from considering the clinician’s charges, Medicare rates, or Medicaid rates. In addition to being considered by arbiters in determining payment, both the insurer’s and clinician’s final offers must be submitted in dollar amounts and as a percentage of the QPA.

Hospital and physician groups have challenged explicit guidance that arbiters select offers closest to the QPA in several lawsuits, and this guidance was vacated by a federal judge in the Eastern District of Texas in February 2022.^3,4,5^ Regulators issued a final rule in August 2022 replacing the QPA presumption with instructions that arbiters must consider the QPA and then also consider the additional allowable information to choose a payment that best represents the value of the item or service under dispute.^6^ However, as the only monetary value shown to arbiters, it may become an anchoring point with or without specific guidance to use it as such.^7,8^

Ancillary physicians (eg, anesthesiologists, pathologists, and radiologists) and physicians who provide emergency care typically have consistent patient volume because demand for their services is emergent or indirectly chosen. Thus, physicians in these specialties do not face the typical trade-off of accepting lower reimbursements in exchange for a higher volume of patients through participation in insurance networks.^9^ These specialties bill out of network more often than other specialties, and emergency physicians collect more on average through out-of-network billing than for in-network care.^10,11^ Thus, payment dispute resolutions that favor offers closer to the QPA may substantially affect out-of-network revenues for emergency and ancillary physicians.

Using the QPA as the benchmark for out-of-network payment disputes will likely affect payment rates that insurers and affected clinicians negotiate for in-network services as well. Although surprise bills were concentrated among a few of these clinicians, higher out-of-network payments spilled over to higher in-network prices because of clinicians’ stronger bargaining position owing to this credible out-of-network option that is unavailable to most other clinicians.^12,13,14^ Previous studies have found that mean contracted rates for most physician specialties are approximately 128% of Medicare rates but are much higher for physicians practicing anesthesiology (344%), emergency medicine (306%), and radiology (200%) who are unconstrained by the price-volume trade-offs of a functioning market.^15,16,17^ Thus, the NSA will not only have implications for the expected payment for out-of-network services but will likely affect negotiated in-network prices by correcting this market failure and shifting the bargaining dynamics. To quantify the potential implications of the NSA for private insurance reimbursements to emergency medicine clinicians, we estimated the QPA for geographic and funding markets and compared 2019 in-network and out-of-network payments with estimates of the QPA calculated from a large multipayer commercial claims data set.

## Methods

### Data Source

In this cross-sectional study, we used 2019 Health Care Cost Institute commercial claims data, comprising claims from Aetna, Humana, and some Blue Health Intelligence group health plans.^18^ This study focused on professional emergency medicine services billed under *Current Procedural Terminology* (*CPT*) codes 99281 through 99285 and 99291. We observed the allowed amount, clinician network status and geographic region, clinician type, insurance product (eg, health maintenance organization, preferred provider organization, point of service, or exclusive provider organization), and funding type (eg, fully insured or self-funded) for each claim. We excluded claims with missing or inconsistent data fields and required at least 500 claims within each market strata underlying any descriptive statistic (eTable 1 in the Supplement). The University of Southern California Institutional Review Board reviewed the study and determined that it met the criteria for coded private information or specimens. This report follows the Strengthening the Reporting of Observational Studies in Epidemiology (STROBE) reporting guideline for cross-sectional studies.

### Estimating Qualifying Payment Amounts

Rulemaking for the NSA defined the QPA as the plan or issuer’s median contracted rate for the same or similar service within a geographic region and insurance market.^2^ Regions are defined as metropolitan statistical areas (MSAs), with a state-level aggregation of all non-MSAs within each state. Qualifying payment amounts are calculated by each carrier separately for individual, small group, and large group markets. Self-funded group health plans can use all plans offered by their sponsor or third-party administrator as their market.

We approximated the QPA service, geographic, and insurance market strata using information available on claims. We used *CPT* codes on claims to define services. We used clinicians’ core-based statistical area information on claims to assign claims to geographic regions of MSAs and state aggregations of non-MSAs. We used the funding status identified on claims (self-funded or fully insured) as proxies for the insurance market because we could not distinguish among issuers in the data set.

We could not observe contracts from the available data, which prohibited us from directly computing the median of contracted rates in the way that the NSA rulemaking defines. Instead, we estimated QPA values for strata defined by *CPT* code, geographic region, and funding type as the median allowed amount of all in-network claims. As a sensitivity analysis, we also estimated the QPA as the median of unique in-network allowed amount values.

### Measurement of QPA vs Mean Allowed Amounts

Within each *CPT* code, geographic region, and funding type stratum, we calculated the ratios of the mean in-network and mean out-of-network allowed amounts to the stratum’s QPA. To produce summary results, we computed volume-weighted means of these ratios across all emergency *CPT* codes within each geographic and funding market stratum. We weighted by the total volume (claim count) of each *CPT* code in the analytic sample. This sample yielded measures comparing mean in-network and out-of-network payments to the QPA for self-funded and fully insured plans in each geographic market. We presented ratios as percentages in the text to enhance interpretation of findings.

Aggregating across *CPT* codes increased the number of claims underlying the descriptive statistic for each geographic and funding market stratum, thus increasing the number of strata meeting the threshold of at least 500 claims to be included in the primary analyses (the threshold was set by the data provider under the data use agreement). As a sensitivity analysis, we replicated descriptive statistics disaggregating by *CPT* code.

### Statistical Analyses

Data were analyzed November 1, 2021, to April 7, 2022. For in-network and out-of-network services, we calculated unweighted means across strata to compute national measures of ratios of mean payment to the estimated QPA. The data were not necessarily representative of the national commercially insured population, so weighting by claims volume could unnecessarily skew our results. This approach essentially equally weighted each stratum. We also stratified descriptive statistics by plan funding type and for physicians and nonphysicians (ie, nurse practitioners and physician assistants). We geographically displayed the ratios of mean in-network and out-of-network allowed amounts to the QPA in a series of maps. These maps showed regional patterns and illustrated the geographic composition of the data. We also computed the proportion of strata with higher mean payments than the estimated QPA. As a sensitivity analysis, we described the ratios of mean in-network and out-of-network allowed amounts to the QPA by funding status among the subset of geographic regions with sufficient data for both network and funding types.

We fit a series of linear regression models to assess how geographic market, geography type (MSAs vs non-MSAs), and funding market accounted for the variation in the ratios of mean in-network and out-of-network allowed amounts to the QPA across strata. The models were fit at the stratum level with the ratio of mean in-network allowed amount to the QPA as the dependent variable in 1 series and the ratio of mean out-of-network allowed amount to the QPA as the dependent variable in a second series. Three models separately used funding status, region type, and region as independent variables. A fourth model included funding status, region type, and region together as independent variables. Models were replicated at the claim level as a sensitivity analysis. All statistical analyses were performed using Stata, version 15 (StataCorp LLC), and a 2-sided *P* = .05 was considered to be statistically significant.

## Results

### Sample

The study included 8 960 691 professional claims for *CPT* codes 99281, 99282, 99283, 99284, 99285, and 99291 for patients younger than age 65 years with no secondary coverage. After applying exclusion criteria (eTable 1 in the Supplement), our analytic sample included 7 556 541 claims.

We compared the QPA estimates with in-network payments in 371 self-funded (325 [87.6%] MSAs; 46 [12.4%] non-MSAs) and 279 fully insured (237 [85.0%] MSAs; 42 [15.1%] non-MSAs) geographic markets. The sample included fewer out-of-network claims, limiting the comparison to 153 self-funded (127 [83.0%] MSAs; 26 [17.0%] non-MSAs) and 74 fully insured (59 [79.7%] MSAs; 15 [20.3%] non-MSAs) geographic markets for those analyses.

Most claims in the sample were for *CPT* codes 99285 (34.9%), 99284 (33.1%), and 99283 (23.0%) (Table 1). In-network claims comprised 87.7% of the sample. Most services occurred in emergency departments (74.4%), with a few in the outpatient (22.5%) and inpatient (3.1%) hospital settings. Claims were predominantly from self-funded plans (71.6%) and preferred provider organization (61.0%) or point of service (29.7%) product types. Physicians rendered 90.1% of the services, and nonphysicians rendered 6.3% of services. Clinician type was unknown in 3.6% of claims.

**Table 1.** Sample Characteristics

Characteristic	Claims, No. (%)
Total sample	7 556 541 (100)
*Current Procedural Terminology* code	
99281	29 040 (0.4)
99282	217 286 (2.9)
99283	1 740 269 (23.0)
99284	2 498 631 (33.1)
99285	2 638 149 (34.9)
99291	433 166 (5.7)
Network status	
In network	6 623 264 (87.7)
Out of network	933 277 (12.4)
Point of service	
Emergency department	5 621 959 (74.4)
Outpatient hospital	1 697 106 (22.5)
Inpatient hospital	237 476 (3.1)
Clinician type	
Physician	6 808 209 (90.1)
Nonphysician	478 669 (6.3)
Unknown	269 663 (3.6)
Geography type	
Metropolitan statistical areas	6 424 886 (85.0)
Non–metropolitan statistical areas (state)	1 131 655 (15.0)
Funding type	
Self-funded	5 411 663 (71.6)
Fully insured	2 144 878 (28.4)
Product	
Preferred provider organization	4 610 802 (61.0)
Point of service	2 246 934 (29.7)
Health maintenance organization	558 258 (7.4)
Exclusive provider organization	140 547 (1.9)
Allowed amount, mean (SD), $	313 (306)
Qualifying payment amount, mean (SD), $	252 (133)

### Comparing QPA Estimates With Mean Payments

**Figure 1.**
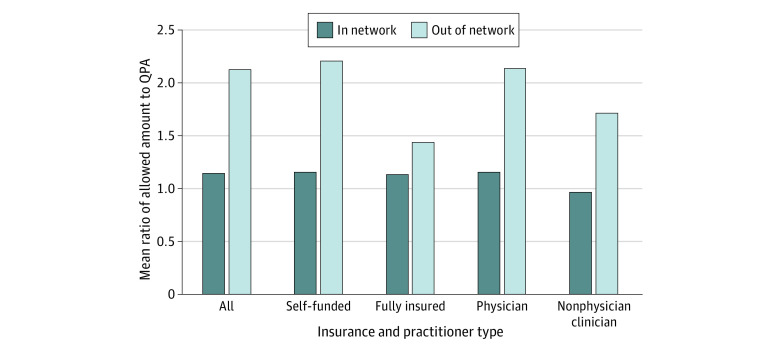
Mean Ratio of Allowed Amount to Estimated Qualifying Payment Amount Across Strata Nonphysicians include nurse practitioners and physician assistants. QPA indicates qualifying payment amount.

Among all claims in the sample, the mean (SD) allowed amount was $313 ($306), and the mean (SD) QPA was $252 ($133). Standardizing by CPT claim volume and averaging across all funding and geographic strata, the mean in-network allowed amounts were 14% (ratio, 1.14) higher than the estimated QPA, and the mean out-of-network allowed amounts were 112% (ratio, 2.12) higher than the estimated QPA (Figure 1).

The distributions of the ratios of mean in-network and out-of-network allowed amounts to the QPA estimates are shown in eFigures 1 and 2 in the Supplement. Mean in-network payments were lower than the QPA estimates in 26% of the sample, with most strata having in-network payments between the QPA and 50% higher than the QPA, and a small number of strata having even higher in-network payments. The distribution of out-of-network payments was higher, with only 13% of strata having mean out-of-network payments lower than their QPA estimates. Some strata had mean out-of-network payments more than 400% higher than their QPAs.

In-network payments were similar among self-funded (15% [ratio, 1.15] greater than QPA estimates) and fully insured (13% [ratio, 1.13] greater than QPA estimates) plans, but out-of-network payments were more generous among self-funded plans (Figure 1). Mean out-of-network payments among self-funded plans were 120% (ratio, 2.20) higher than the QPA estimate but only 43% (ratio, 1.43) higher than the QPA for fully insured plans. These patterns were consistent in a sensitivity analysis of the ratios of mean in-network and out-of-network allowed amounts to the QPA estimates by funding status among the subset of 73 geographic regions with sufficient data for both network and funding types (eAppendix 1 and eFigure 3 in the Supplement).

Mean payments for physicians were 15% (ratio, 1.15) higher than the QPA estimate when in network and 113% (ratio, 2.13) higher than the QPA when out of network. In contrast, nonphysicians’ mean in-network allowed amounts were 4% (ratio, 0.96) lower than the QPA estimate, and their mean out-of-network payments were 71% (ratio, 1.71) higher than the QPA estimate.

**Figure 2.**
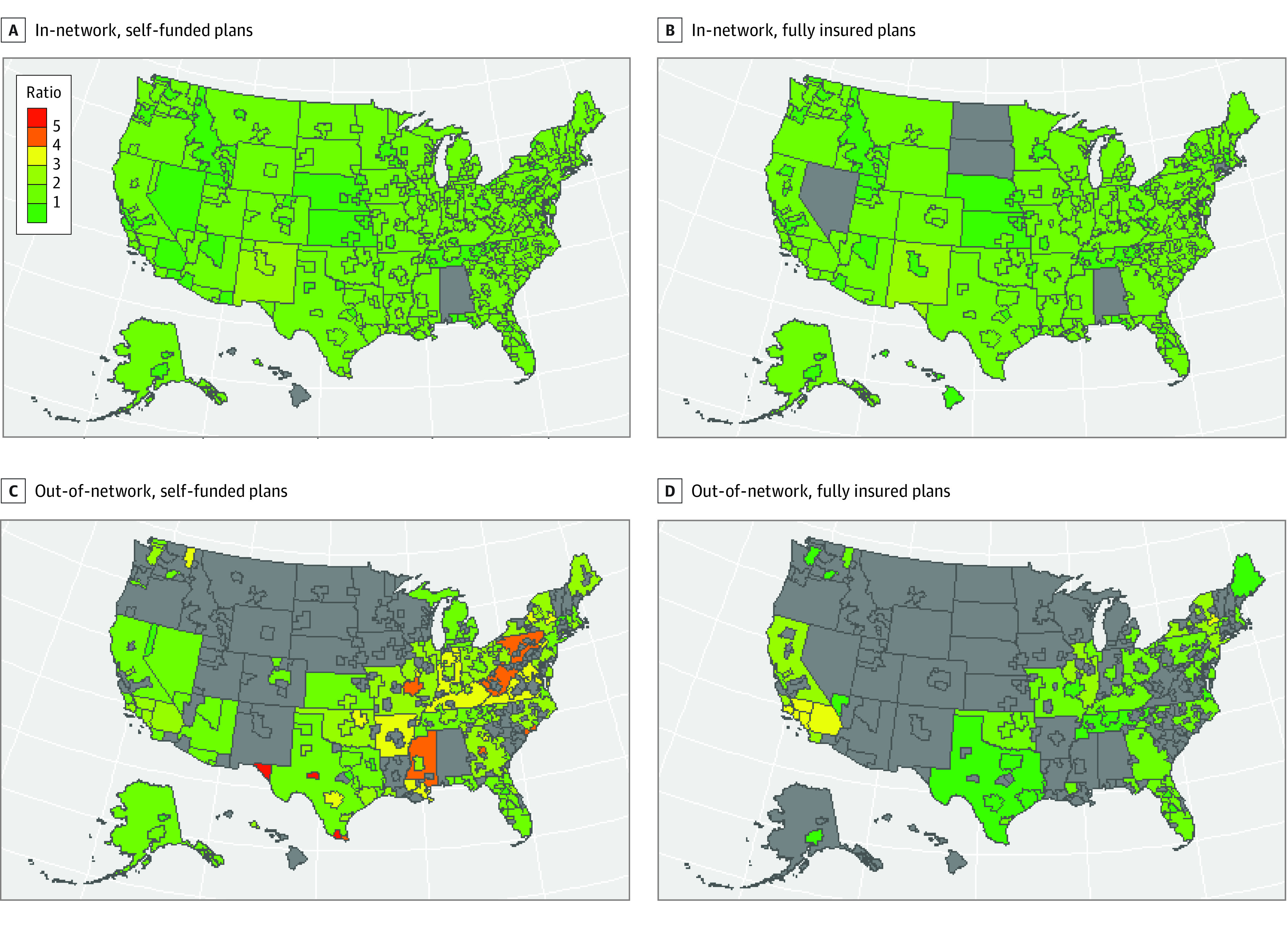
Ratio of Allowed Amounts to Estimated Qualifying Payment Amount Gray indicates that information was not available for that area.

We observed similar geographic patterns in the magnitude of in-network payments to the estimated QPA between self-funded and fully insured plans (Figure 2A and B). For example, in all geographic strata across self-funded and fully insured plans, ratios of in-network allowed amounts to estimated QPA were below 300%, and most were below 200%. In contrast, we observed deviations between self-funded and fully insured plans for out-of-network payments relative to the estimated QPA within many regions (Figure 2C and D). For example, few markets exhibit ratios of out-of-network allowed amounts to estimated QPA above 300% for fully insured plans. However, we estimate that self-insured plans have out-of-network allowed amounts in excess of 400% of estimated QPA in numerous markets. In regression models, geographic region accounted for the most variation in the ratios of in-network and out-of-network allowed amounts to the QPA estimates, as indicated by the high *R*^2^ values for models, including the region fixed-effects model (Table 2). Self-funded plans were associated with ratios of mean out-of-network allowed amounts to the QPA estimate that were 68.2 percentage points higher than fully insured plans (*P* < .001), adjusting for region and region type.

**Table 2.** Regression of Relative Magnitudes of Payments and Estimated QPA With Plan Characteristics and Geography

Characteristic	Percentage point change in ratio of mean in-network allowed amount to QPA (n = 650)^a^						Percentage point change in ratio of mean out-of-network allowed amount to QPA (n = 227)^a^					
	Adjusted for plan type only	*P* value	Adjusted for MSAs only	*P* value	Adjusted for plan type, MSAs, and region^b^	*P* value	Adjusted for plan type only	*P* value	Adjusted for MSAs only	*P* value	Adjusted for plan type, MSAs, and region^b^	*P* value
Self-funded	1.7	.37	NA	NA	2.2	.10	76.3	<.001	NA	NA	68.2	<.001
Non-MSAs	NA	NA	7.3	.01	11.5	.47	NA	NA	8.6	.62	−3.2	.73
Constant	113.1	<.001	113.1	<.001	87.5	<.001	143.3	<.001	193.2	<.001	71.4	.28
*R* ^2^	0.001	NA	0.011	NA	0.817	NA	0.13	NA	0.001	NA	0.864	NA

Abbreviations: MSAs, metropolitan statistical areas; QPA, qualifying payment amount.

^a^ In each model, individual claims are aggregated to the geographic market and funding strata level so that each observation outcome is the ratio of mean allowed amounts to the estimated strata-level QPA. Strata with at least 500 claims for a given network status (in-network or out-of-network payment) are included.

^b^ Model includes region fixed effects.

We observed similar results between primary results and 2 sensitivity analyses results by disaggregating by *CPT* code (eTables 2 and 3 in the Supplement) and using an alternative estimate of the QPA measured as the median of unique in-network allowed amount values (eAppendix 2, eFigures 4-11, and eTable 4 in the Supplement). For example, using a sample of only claims with the *CPT* code 99285, we estimated that in-network allowed amounts for fully insured plans were 13% (ratio, 1.13) higher than estimated QPA and 14% (ratio, 1.14) higher for self-funded plans. Out-of-network allowed amounts were 53% (ratio, 1.53) higher than estimated QPA for fully insured plans and 128% (ratio, 2.28) higher for self-funded plans. Regression models fit at the claim level yielded similar results to models fit at the strata level (eTable 5 in the Supplement).

## Discussion

This claims-based study estimated the QPAs associated with implementation of the NSA and compared these estimates with mean in-network and out-of-network allowed amounts for professional emergency medicine services before NSA implementation. We estimated QPA values lower than mean in-network and out-of-network allowed amounts in most of the geographic regions and funding strata in the sample. In particular, we estimated that self-funded plans’ mean out-of-network payments were nearly double the QPA estimate. We also found substantial geographic heterogeneity in the ratios of in-network and out-of-network payments to the QPA.

The positive skew of the in-network distributions is due to large outlier in-network payments, which may reflect the relative bargaining sophistication and market power of some emergency physician staffing companies.^12^ Because the QPA is defined as the median in-network payment, if in-network payments converge to the QPA, roughly half of in-network payments will decrease and half will increase. However, with positively skewed in-network payment distributions in most cases (whereby the mean payment is higher than the median), in-network reimbursements would fall in the aggregate. Nonphysician emergency clinicians may not experience this downward payment pressure because their mean in-network allowed amounts were just below the QPA estimate. This difference between physicians and nonphysicians in-network payments is likely because many insurers pay nonphysicians less than physicians for the same services.^19^

Mean out-of-network payments were 112% higher than the QPA and in some cases more than 5 times higher than the QPA. Furthermore, this calculation does not include any additional revenue out-of-network clinicians earn from balance billing. This disparity may reflect the pre-NSA strategy of some physician staffing companies to exit contracts with insurers and set high charges.^12^ This may have been a lucrative strategy, particularly when treating patients with self-funded health plans given the often generous out-of-network reimbursement of self-funded plans.^13,20^ If payments converge to the QPA, the out-of-network revenue earned by emergency medicine clinicians would be reduced, in many cases substantially. In particular, out-of-network payments made by self-funded health plans may decline sharply.

### Limitations

This study has limitations. We could not distinguish among the 3 insurance payers in the sample, and we did not directly observe contracts. Rulemaking defining the QPA specifies that it should be based on contracts held by individual carriers or plan sponsors; thus, our approaches to QPA estimation were deviations from the prescribed QPA methodology. Furthermore, the payers in our sample may not be representative of the entire commercial market. The data set did not include any nongroup health plans, and we could not distinguish between small and large group plans. We presented only descriptive statistics with more than 500 underlying claims, limiting the *CPT* code granularity and geographic breadth of the analyses. Figure 2 displays the geographic data coverage and shows inconsistent coverage across regions.

## Conclusions

In this cross-sectional study of US commercial insurance claims, mean 2019 in-network and out-of-network payments were 14% and 112% above the estimated QPA, respectively. Mean out-of-network payments were higher among self-funded plans than fully insured plans and higher among physicians than nonphysicians. Before the NSA, emergency medicine physicians outside of patients’ insurance networks often received insurer reimbursement exceeding median in-network rates and additional out-of-pocket payments from patients through surprise balance bills. Clinicians in these specialties had superior bargaining power for in-network payments because their patient volume did not depend on network status. Surprise billing for out-of-network services affected negotiations for in-network rates through the threat that, by not contracting with emergency clinicians at inflated rates, patients might be exposed to large surprise bills. The NSA eliminates the possibility that emergency medicine clinicians can extract these additional payments from patients. Results of the present study suggest that using the QPA as the benchmark for out-of-network payment disputes will likely exert broad downward pressure on professional emergency medicine payments that partially corrects for upward price pressure under the bargaining environment before the NSA.

## Notes

Supplement. **eTable 1.** Attrition Table With Steps Applied Sequentially **eFigure 1.** In-Network Distribution of Ratios of Allowed Amounts to Estimated Qualifying Payment Amount **eFigure 2.** Out-of-Network Distribution of Ratios of Allowed Amounts to Estimated Qualifying Payment Amount **eAppendix 1.** Regional Data Completion **eFigure 3.** Mean Ratios of Allowed Amount to Qualifying Payment Amount Among a Consistent Set of 73 Regions With Complete Data for All Network and Funding Strata **eTable 2.** Summary of Mean In-Network Allowed Amount, Estimated Qualifying Payment Amount, and Ratio of In-Network Allowed Amount to Estimated Qualifying Payment Amount Across Strata **eTable 3.** Summary of Mean Out-of-Network Allowed Amount, Estimated Qualifying Payment Amount, and Ratio of Out-of-Network Allowed Amount to Estimated Qualifying Payment Amount Across Strata **eAppendix 2.** QPA Values for Strata Defined by *Current Procedural Terminology*, Geographic Region, and Funding Type **eFigure 4.** Box Plot of Qualifying Payment Amount Using 2 Measurement Methods **eFigure 5.** Mean Ratio of Allowed Amount to Qualifying Payment Amount Across Strata **eFigure 6.** Histogram of Ratios of Mean In-Network Allowed Amount to Qualifying Payment Amount **eFigure 7.** Histogram of Ratios of Mean Out-of-Network Allowed Amount to Qualifying Payment Amount **eTable 4.** Regression Results **eFigure 8.** Ratio of In-Network Mean Allowed Amount to Qualifying Payment Amount Among Self-Funded Plans **eFigure 9.** Ratio of In-Network Mean Allowed Amount to Qualifying Payment Amount Among Fully Insured Plans **eFigure 10.** Ratio of In-Network Mean Allowed Amount to Qualifying Payment Amount Among Self-Funded Plans **eFigure 11.** Ratio of Out-of-Network Mean Allowed Amount to Qualifying Payment Amount Among Fully Insured Plans **eTable 5.** Claim Level Regression Results
