# Supplementary material for: Comparison of Estimated No Surprises Act Qualifying Payment Amounts and Payments to In-Network and Out-of-Network Emergency Medicine Professionals
Source: JAMA Health Forum. 2022 Sep 16;3(9):e223085. doi: 10.1001/jamahealthforum.2022.3085 (PMC9482054; doi:10.1001/jamahealthforum.2022.3085)
Supplement: Supplement. — eTable 1. Attrition Table With Steps Applied Sequentially eFigure 1. In-Network Distribution of Ratios of Allowed Amounts to Estimated Qualifying Payment Amount eFigure 2. Out-of-Network Distribution of Ratios of Allowed Amounts to Estimated Qualifying Payment Amount eAppendix 1. Regional Data Completion eFigure 3. Mean Ratios of Allowed Amount to Qualifying Payment Amount Among a Consistent Set of 73 Regions With Complete Data for All Network and Funding Strata eTable 2. Summary of Mean In-Network Allowed Amount, Estimated Qualifying Payment Amount, and Ratio of In-Network Allowed Amount to Estimated Qualifying Payment Amount Across Strata eTable 3. Summary of Mean Out-of-Network Allowed Amount, Estimated Qualifying Payment Amount, and Ratio of Out-of-Network Allowed Amount to Estimated Qualifying Payment Amount Across Strata eAppendix 2. QPA Values for Strata Defined by Current Procedural Terminology, Geographic Region, and Funding Type eFigure 4. Box Plot of Qualifying Payment Amount Using 2 Measurement Methods eFigure 5. Mean Ratio of Allowed Amount to Qualifying Payment Amount Across Strata eFigure 6. Histogram of Ratios of Mean In-Network Allowed Amount to Qualifying Payment Amount eFigure 7. Histogram of Ratios of Mean Out-of-Network Allowed Amount to Qualifying Payment Amount eTable 4. Regression Results eFigure 8. Ratio of In-Network Mean Allowed Amount to Qualifying Payment Amount Among Self-Funded Plans eFigure 9. Ratio of In-Network Mean Allowed Amount to Qualifying Payment Amount Among Fully Insured Plans eFigure 10. Ratio of In-Network Mean Allowed Amount to Qualifying Payment Amount Among Self-Funded Plans eFigure 11. Ratio of Out-of-Network Mean Allowed Amount to Qualifying Payment Amount Among Fully Insured Plans eTable 5. Claim Level Regression Results [file jamahealthforum-e223085-s001.pdf]

Supplementary Online Content

Duffy EL, Biener A, Garmon C, Trish EE. Comparison of estimated No Surprises Act qualifying payment amounts and payments to in-network and out-of-network emergency medicine professionals. *JAMA Health Forum*. 2022;3(9):e223085. doi:10.1001/jamahealthforum.2022.3085

eTable 1. Attrition Table With Steps Applied Sequentially
eFigure 1. In-Network Distribution of Ratios of Allowed Amounts to Estimated Qualifying Payment Amount
eFigure 2. Out-of-Network Distribution of Ratios of Allowed Amounts to Estimated Qualifying Payment Amount
eAppendix 1. Regional Data Completion
eFigure 3. Mean Ratios of Allowed Amount to Qualifying Payment Amount Among a Consistent Set of 73 Regions With Complete Data for All Network and Funding Strata
eTable 2. Summary of Mean In-Network Allowed Amount, Estimated Qualifying Payment Amount, and Ratio of In-Network Allowed Amount to Estimated Qualifying Payment Amount Across Strata
eTable 3. Summary of Mean Out-of-Network Allowed Amount, Estimated Qualifying Payment Amount, and Ratio of Out-of-Network Allowed Amount to Estimated Qualifying Payment Amount Across Strata
eAppendix 2. QPA Values for Strata Defined by *Current Procedural Terminology*, Geographic Region, and Funding Type
eFigure 4. Box Plot of Qualifying Payment Amount Using 2 Measurement Methods
eFigure 5. Mean Ratio of Allowed Amount to Qualifying Payment Amount Across Strata
eFigure 6. Histogram of Ratios of Mean In-Network Allowed Amount to Qualifying Payment Amount
eFigure 7. Histogram of Ratios of Mean Out-of-Network Allowed Amount to Qualifying Payment Amount
eTable 4. Regression Results
eFigure 8. Ratio of In-Network Mean Allowed Amount to Qualifying Payment Amount Among Self-Funded Plans
eFigure 9. Ratio of In-Network Mean Allowed Amount to Qualifying Payment Amount Among Fully Insured Plans
eFigure 10. Ratio of In-Network Mean Allowed Amount to Qualifying Payment Amount Among Self-Funded Plans
eFigure 11. Ratio of Out-of-Network Mean Allowed Amount to Qualifying Payment Amount Among Fully Insured Plans
eTable 5. Claim Level Regression Results

This supplementary material has been provided by the authors to give readers additional information about their work.

© 2022 Duffy EL et al. *JAMA Health Forum*.

eTable 1. Attrition Table With Steps Applied Sequentially

	N observations remaining	% Observations from starting sample remaining	N observations dropped in each step	% Observations dropped in each step
Starting sample of claim lines	8,960,691	100.0%		
Remove claims with multiple claim lines	8,703,816	97.1%	256,875	2.9%
Remove claims for patients with multiple insurance records for the month of the service	8,682,280	96.9%	21,536	0.2%
Remove if units not equal to one	8,593,874	95.9%	88,406	1.0%
Remove if allowed amount is zero or negative	8,231,232	91.9%	362,642	4.0%
Remove if network status is missing	8,177,060	91.3%	54,172	0.6%
Remove indemnity and unknown plan type	8,158,674	91.0%	18,386	0.2%
Remove is POS is not 21 (inpatient), 22 (outpatient), 23 (ED)	8,152,765	91.0%	5,909	0.1%
Remove if provider state is missing	7,728,980	86.3%	423,785	4.7%
Remove if state is Alabama to comply with data contributor masking requirements	7,679,162	85.7%	49,818	0.6%
Remove strata with fewer than 500 claims	7,556,541	84.3%	122,621	1.4%

© 2022 Duffy EL et al. *JAMA Health Forum*.

eFigure 1. In-Network Distribution of Ratios of Allowed Amounts to Estimated Qualifying Payment Amount

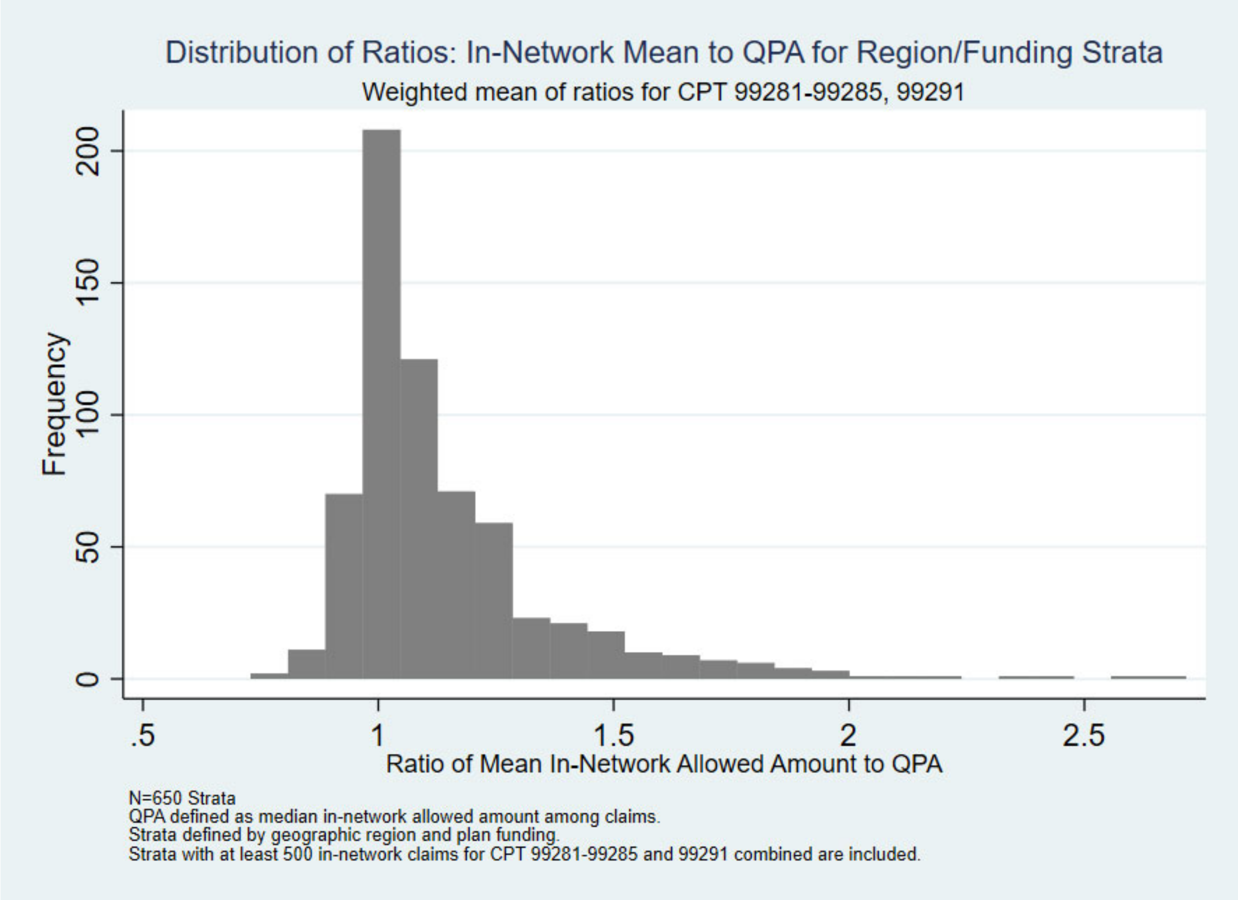

© 2022 Duffy EL et al. *JAMA Health Forum*.

eFigure 2. Out-of-Network Distribution of Ratios of Allowed Amounts to Estimated Qualifying Payment Amount

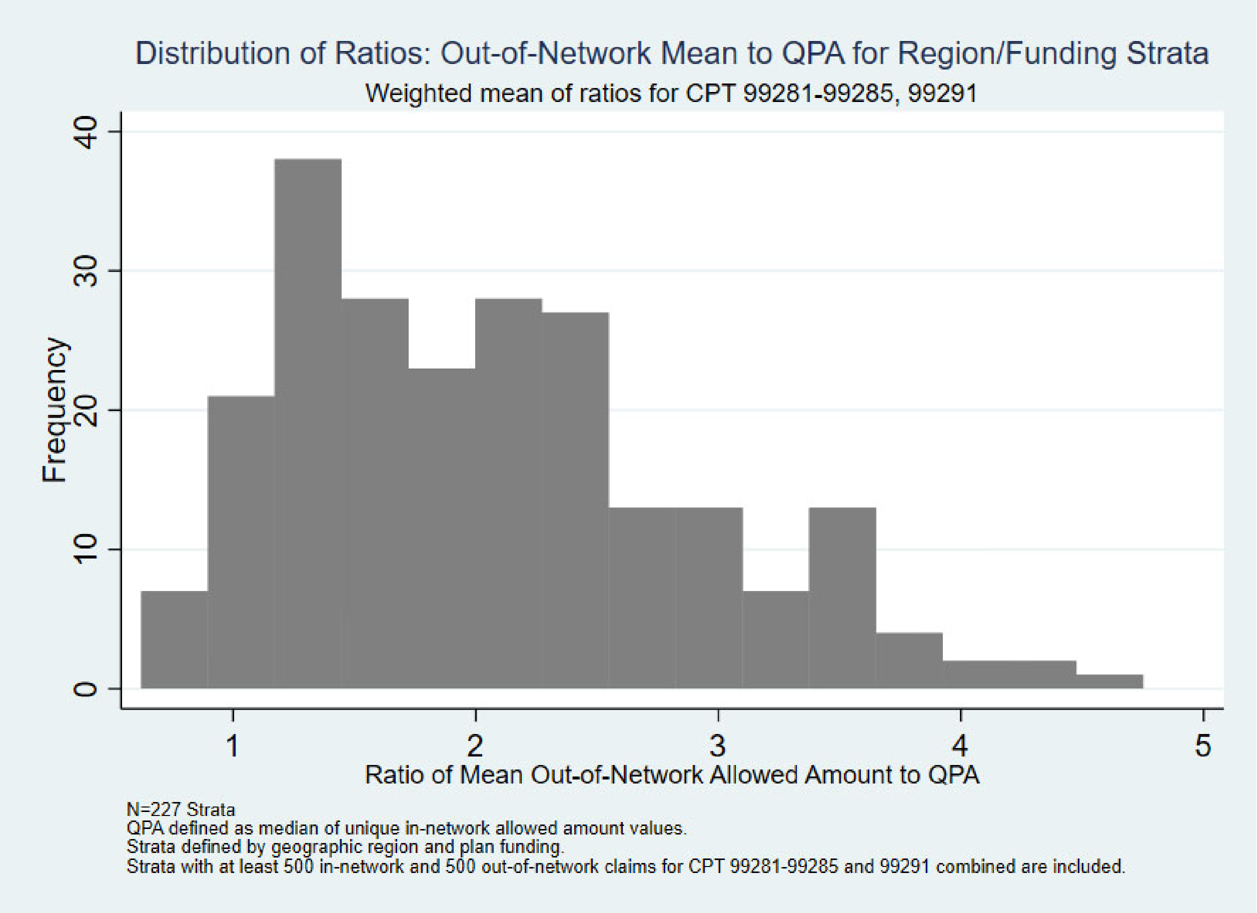

© 2022 Duffy EL et al. *JAMA Health Forum*.

eAppendix 1. Regional Data Completion

In our analytic sample, regional data completion varies for in- and out-of-network claims among self-funded and fully-insured plans. This is due to variation in the market coverage of the insurance carriers contributing claims to the Health care Cost Institute commercial claims database. This inconsistent regional composition underlying the ratios of mean in-network and out-of-network allowed amounts to QPA by funding status could bias our comparisons. To address this concern, we use multivariate regression models adjusting for region. We have also conducted a sensitivity analysis describing the ratios of mean in-network and out-of-network allowed amounts to QPA by funding status among the subset of geographic regions with sufficient data for both network and funding types

We have identified 73 regions with sufficient data for in-network and out-of-network analyses among both fully-insured and self-funded plans. The mean ratios of allowed amount to QPA among this subset of regions are shown below in eFigure 3. These results are similar to those observed among the full analytic sample, as presented in Figure 1.

© 2022 Duffy EL et al. *JAMA Health Forum*.

eFigure 3. Mean ratios of allowed amount to qualifying payment amount among a consistent set of 73 regions with complete data for all network and funding strata

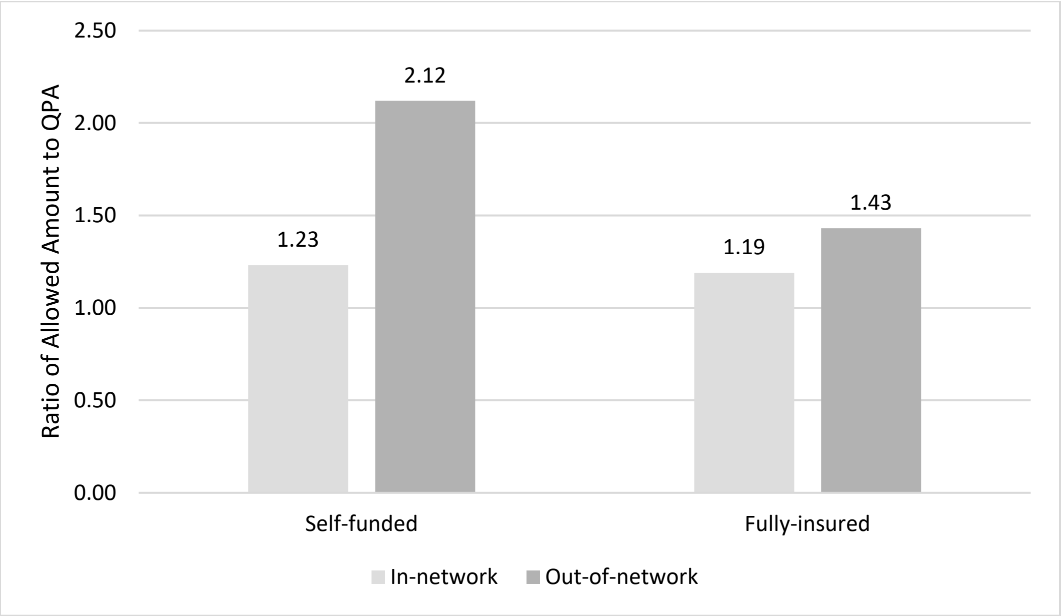

© 2022 Duffy EL et al. *JAMA Health Forum*.

eTable 2. Summary of Mean In-network Allowed Amount, Estimated Qualifying Payment Amount, and Ratio of In-network Allowed Amount to Estimated Qualifying Payment Amount across Strata

Funding and CPT	N Strata	Mean in-network allowed amount across strata	Mean QPA across strata	Mean of strata-level ratios of in-network allowed amount to QPA
Self-funded				
**99281**	3	$66.11	$39.18	1.78
**99282**	72	$84.57	$69.66	1.22
**99283**	240	$143.52	$121.84	1.21
**99284**	281	$252.38	$225.34	1.15
**99285**	266	$384.16	$349.92	1.14
**99291**	98	$431.03	$386.90	1.13
**Aggregated CPTs**	371	$283.14	$256.59	1.15
Fully-insured				
**99282**	28	$83.66	$72.34	1.18
**99283**	142	$140.22	$123.40	1.17
**99284**	176	$246.65	$224.85	1.12
**99285**	168	$373.33	$334.82	1.13
**99291**	50	$424.60	$376.82	1.14
**Aggregated CPTs**	279	$276.80	$253.74	1.13

© 2022 Duffy EL et al. *JAMA Health Forum*.

eTable 3. Summary of Mean Out-of-network Allowed Amount, Estimated Qualifying Payment Amount, and Ratio of Out-of-network Allowed Amount to Estimated Qualifying Payment Amount across Strata

Funding and CPT	N Strata	Mean out-of-network allowed amount across strata	Mean QPA across strata	Mean of strata-level ratios of out-of-network allowed amount to QPA
Self-funded				
**99282**	6	$186.47	$66.07	2.79
**99283**	59	$274.49	$135.46	2.15
**99284**	76	$453.34	$250.35	2.03
**99285**	82	$741.31	$367.07	2.28
**99291**	13	$767.27	$344.33	2.30
**Aggregated CPTs**	153	$508.02	$265.73	2.20
Fully-insured				
**99283**	27	$178.70	$144.45	1.47
**99284**	31	$306.90	$254.71	1.44
**99285**	37	$457.81	$351.95	1.53
**99291**	7	$518.62	$361.46	1.47
**Aggregated CPTs**	74	$324.86	$272.67	1.43

© 2022 Duffy EL et al. *JAMA Health Forum*.

eAppendix 2. QPA Values for Strata Defined by *Current Procedural Terminology*, Geographic Region, and Funding Type

We estimated QPA values for strata defined by CPT, geographic region, and funding type using two approaches:

1. The median allowed amount of all in-network claims
2. The median of unique in-network allowed amount values

The resulting QPA estimates were highly correlated (rho=0.77). eFigure 4 displays the distribution of each QPA estimate by CPT code.

We reproduced our main exhibits using the alternative approach to estimating QPA. QPA was calculated as the median of unique allowed amounts, building on an assumption that unique allowed amount values may proxy contracts.

Taking the mean across strata, we observe that mean in-network allowed amounts are 24% higher than QPA and mean out-of-network allowed amounts are 121% higher than estimated QPA (eFigure 5). The two histograms that follow display the distributions of the ratios of mean in-network and out-of-network allowed amounts to QPA (eFigures 6-7).

Our regression finding holds that region accounts for the majority of variation in the ratios of in-network and out-of-network allowed amounts to QPA (eTable 4). We also continue to find that self-funded plans have higher ratios of mean out-of-network allowed amounts to QPA than fully-insured plans, and this holds when adjusting for region and region type.

Additionally, geographic patterns mapped in eFigures 8-11 are visually similar to our primary analysis (Figure 2).

© 2022 Duffy EL et al. *JAMA Health Forum*.

eFigure 4. Box plot of qualifying payment amount using 2 measurement methods

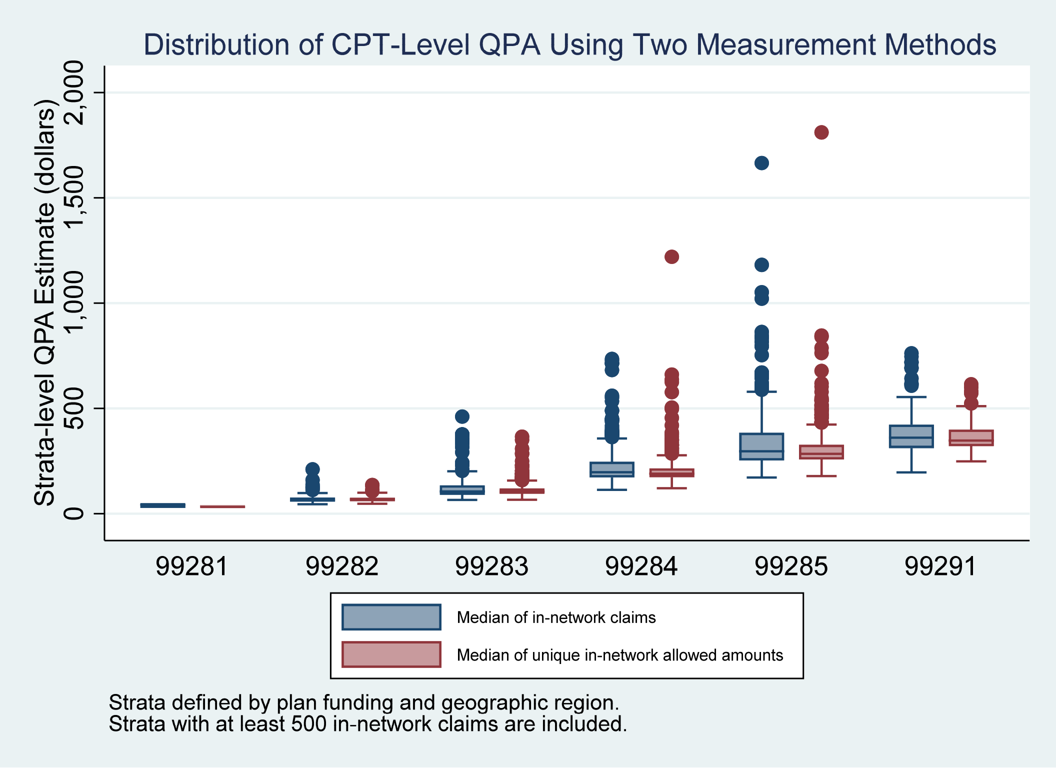

© 2022 Duffy EL et al. *JAMA Health Forum*.

eFigure 5. Mean Ratio of Allowed Amount to Qualifying Payment Amount across Strata

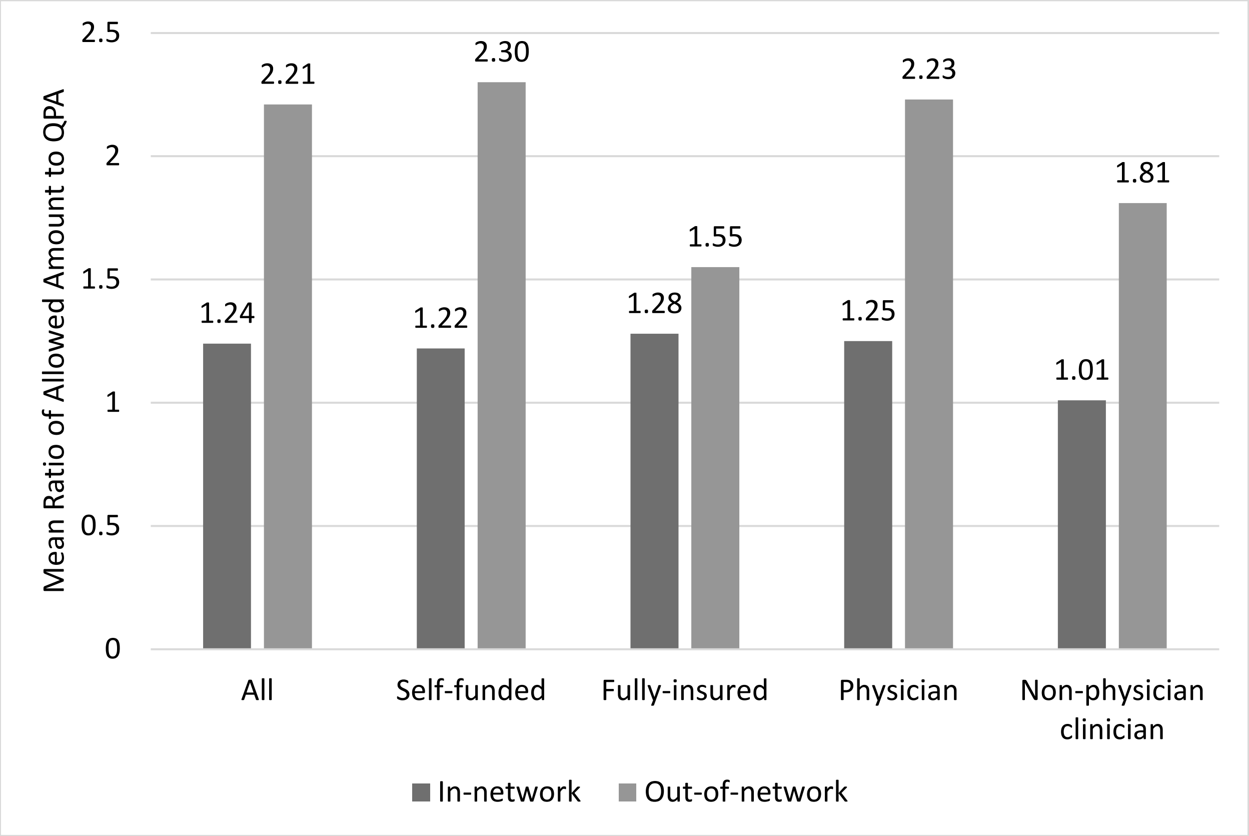

© 2022 Duffy EL et al. *JAMA Health Forum*.

eFigure 6. Histogram of ratios of mean in-network allowed amount to qualifying payment amount

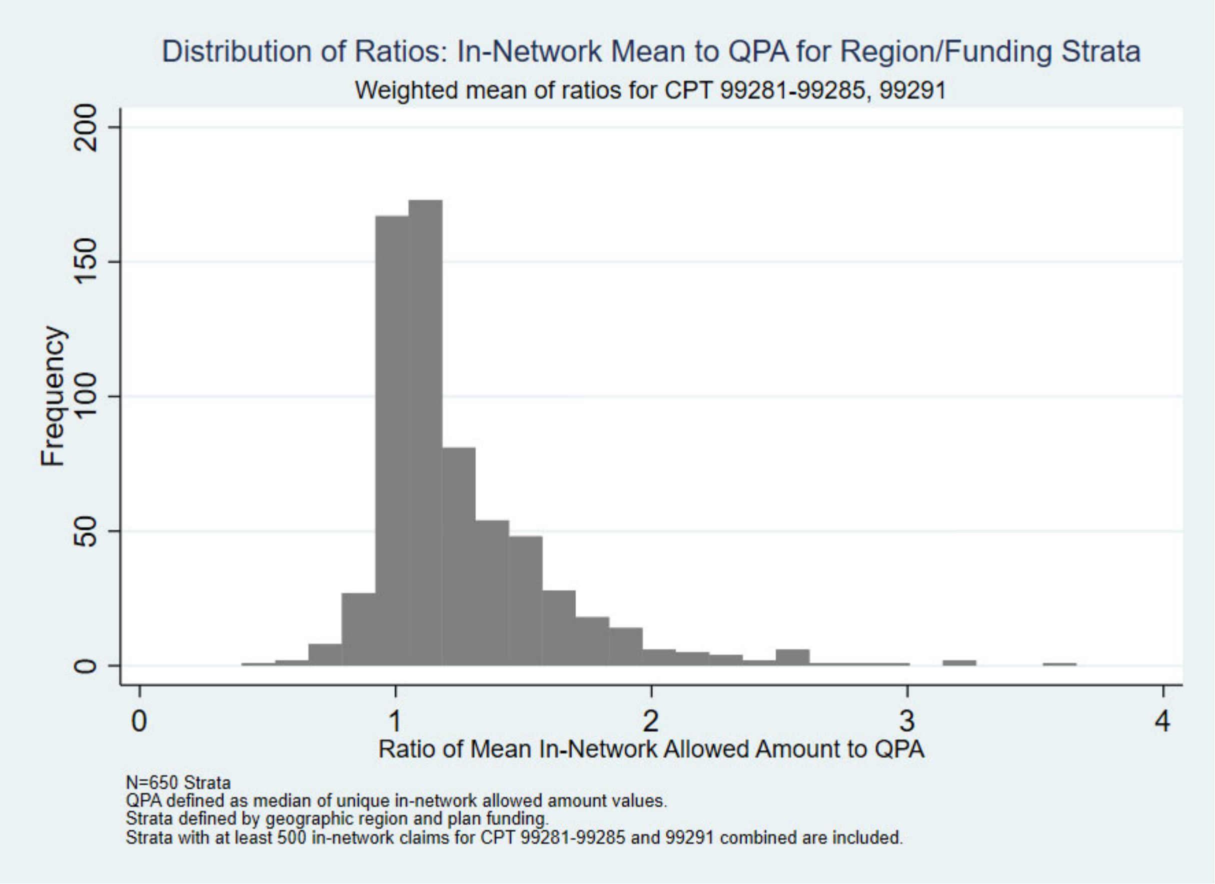

© 2022 Duffy EL et al. *JAMA Health Forum*.

eFigure 7. Histogram of ratios of mean out-of-network allowed amount to qualifying payment amount

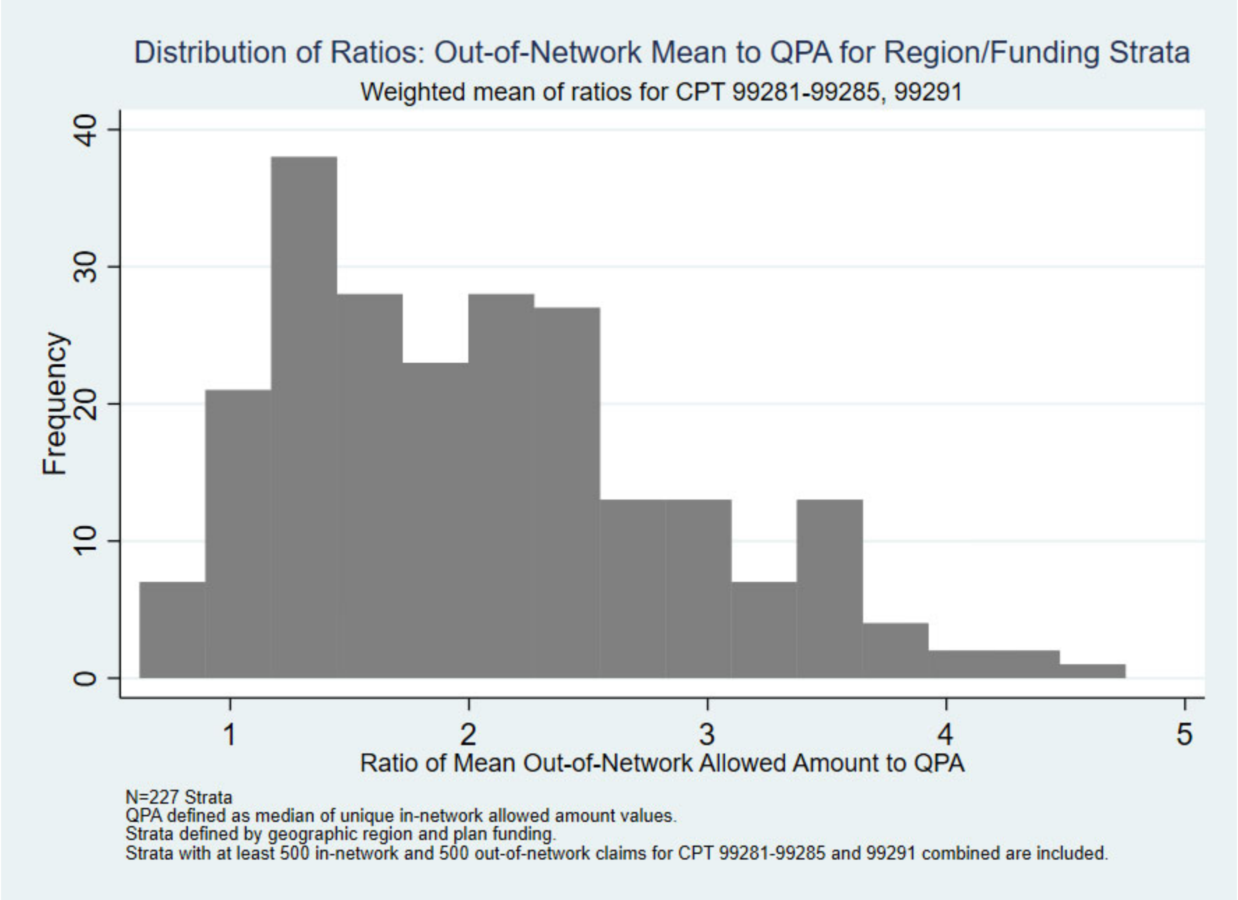

© 2022 Duffy EL et al. *JAMA Health Forum*.

eTable 4. Regression Results

	(1)	(2)	(3)	(4)
Panel A. Ratio of mean in-network allowed amount to QPA				
Observations	650	650	650	650
R-squared	0.006	0.001	0.898	0.900
Est.				
Self-funded	-0.0584**			-0.0372**
Non-MSA Region		-0.0265		0.0543
Control for region	No	No	Yes	Yes
Constant	1.281***	1.251***	1.349***	1.313***
Panel B. Ratio of mean out-of-network allowed amount to QPA				
Observations	227	227	227	227
R-squared	0.183	0.000	0.710	0.845
Est.				
Self-funded	0.751***			0.754***
Non-MSA Region		-0.00177		0.455
Control for region	No	No	Yes	Yes
Constant	1.546***	2.053***	1.805**	0.596

*** p<0.01, ** p<0.05, * p<0.1
Categorical independent variables compare self-funded (versus fully-insured), non-MSA geography (versus MSA).

© 2022 Duffy EL et al. *JAMA Health Forum*.

eFigure 8. Ratio of in-network mean allowed amount to qualifying payment amount among self-funded plans

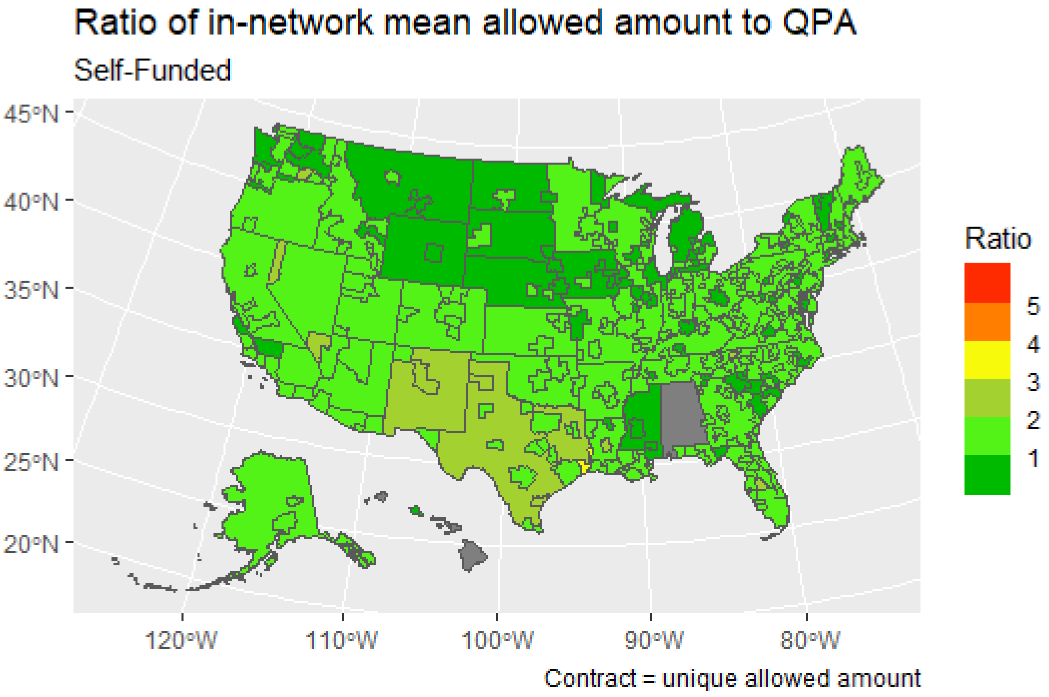

© 2022 Duffy EL et al. *JAMA Health Forum*.

eFigure 9. Ratio of in-network mean allowed amount to qualifying payment amount among fully insured plans

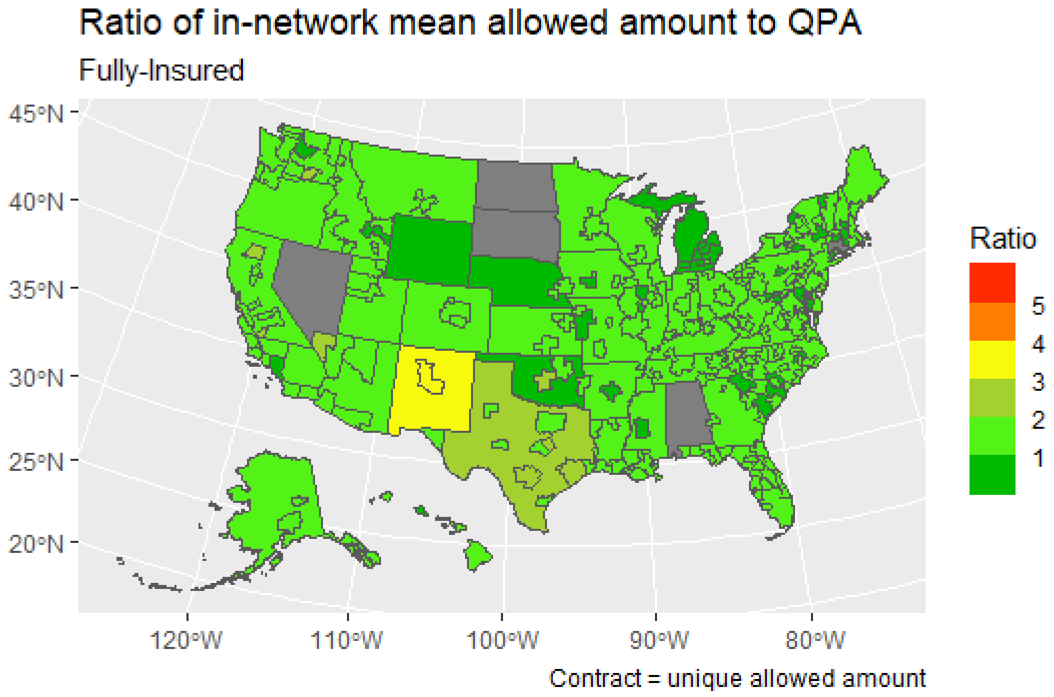

© 2022 Duffy EL et al. *JAMA Health Forum*.

eFigure 10. Ratio of in-network mean allowed amount to qualifying payment amount among self-funded plans

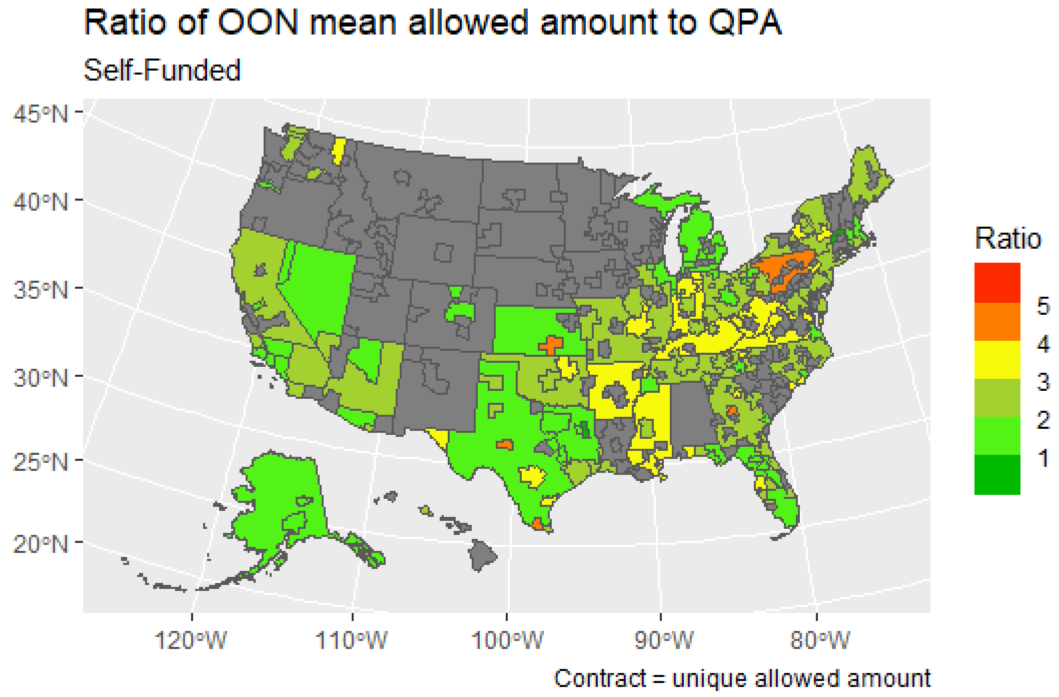

© 2022 Duffy EL et al. *JAMA Health Forum*.

eFigure 11. Ratio of out-of-network mean allowed amount to qualifying payment amount among fully insured plans

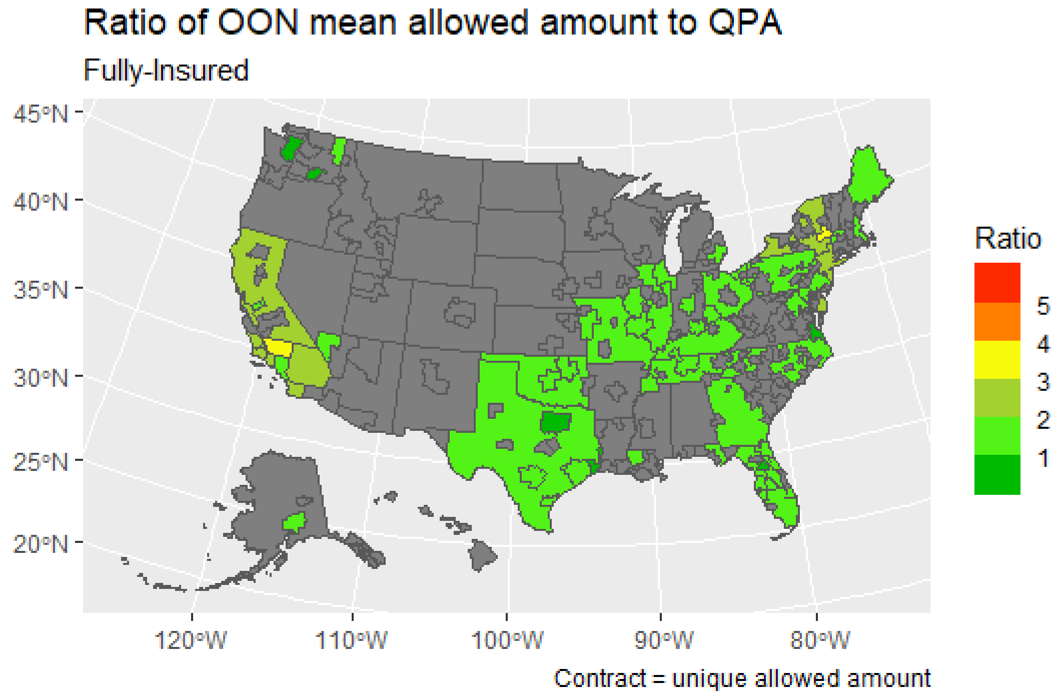

© 2022 Duffy EL et al. *JAMA Health Forum*.

eTable 5. Claim level Regression Results

	(1)	(2)	(3)	(4)	(5)	(6)
Panel A. Ratio of mean in-network allowed amount to QPA						
Observations	6,623,264	6,623,264	6,623,264	6,623,264	6,623,264	6,623,264
R-squared	0.003	0.000	0.002	0.047	0.049	0.055
Est.						
Non-physician	-0.209***				-0.191***	-0.202***
Self-funded		0.0176***			0.0212***	-0.0113***
MSA Region			-0.109***		-0.0837***	-0.0166
Control for region	No	No	No	Yes	Yes	Yes
Control for product and CPT	No	No	No	No	No	Yes
Constant	1.209***	1.184***	1.289***	1.037***	1.017***	0.957***
Panel B. Ratio of mean out-of-network allowed amount to QPA						
Observations	933,277	933,277	933,277	933,277	933,277	933,277
R-squared	0.003	0.015	0.000	0.100	0.117	0.124
Est.						
Non-physician	-0.459***				-0.450***	-0.464***
Self-funded		0.608***			0.652***	0.634***
MSA Region			-0.0139**		0.315***	0.596***
Control for region	No	No	No	Yes	Yes	Yes
Control for product and CPT	No	No	No	No	No	Yes
Constant	1.909***	1.450***	1.926***	1.162***	0.448***	0.457***

*** $p<0.01$, ** $p<0.05$, * $p<0.1$

Note: Models were fit at the claim level with the ratio of mean in-network allowed amount to QPA as the dependent variable in one series and the ratio of mean out-of-network allowed amount to QPA as the dependent variable in a second series. Models 1, 2, 3 and 4 are univariate models with clinician type, funding status, region type, and region as the independent variables, respectively. Model 5 includes clinician type, funding status, region type, and region together as independent variables. Model 6 adds the covariates product type and CPT code as additional covariates. Categorical independent variables compare non-physician clinician (versus physician; unknown clinician not shown), self-funded (versus fully-insured), MSA geography (versus non-MSA).

© 2022 Duffy EL et al. *JAMA Health Forum*.
